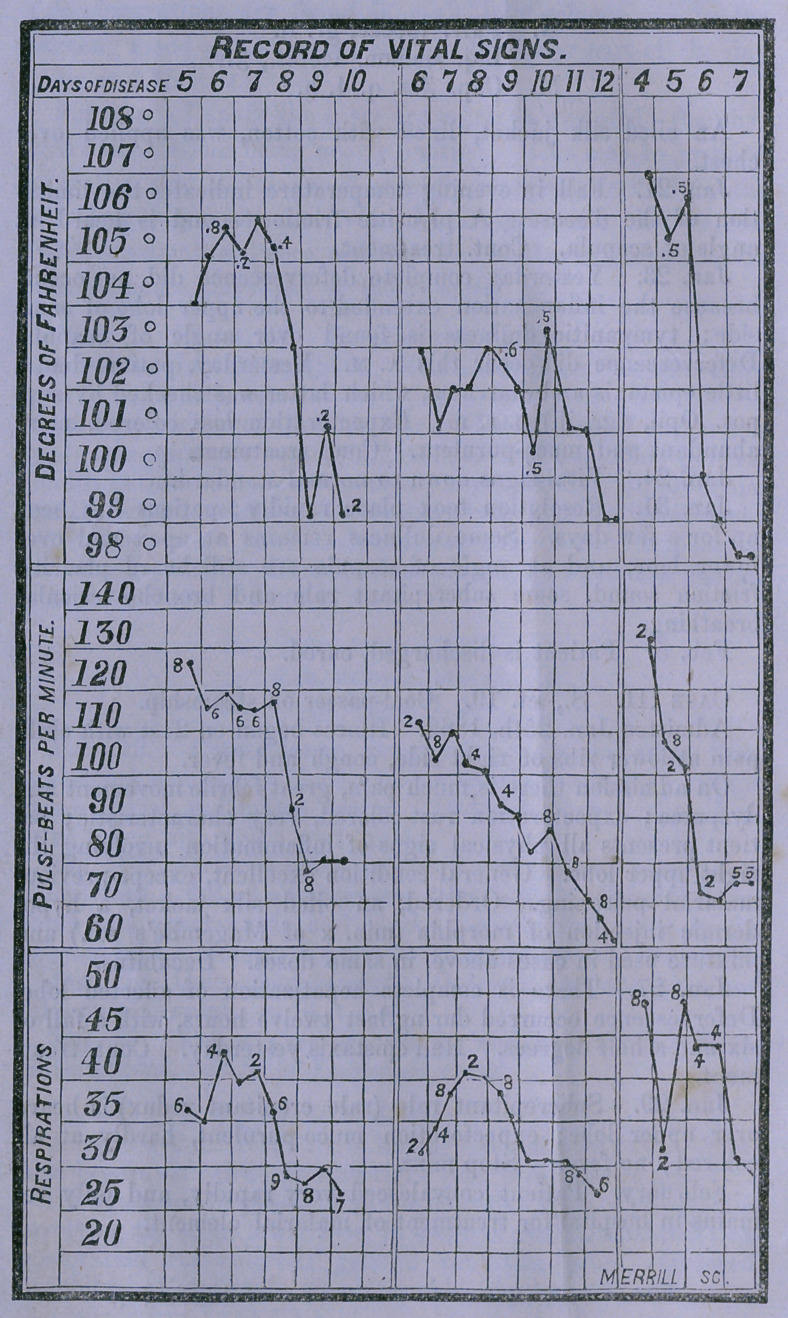# The Use of the Thermometer in Clinical Medicine

**Published:** 1866-05

**Authors:** E. C. Seguin

**Affiliations:** New York City


### CHICAGO MEDICAL JOURNAL.

VOL. XXIII. MAY, 1866. No. 5.

### ORIGINAL CONTRIBUTIONS.

### *The Use of the Thermometer in Clinical Medicine.* By E. C. SEGUIN, M. D., of New York City.

Believing that the matter may prove of interest, and that the attention of practitioners may thus be called to a means of diagnosis and prognosis not second in importance to any single one hitherto employed, the following cases of pneumonia, observed and treated in the New York Hospital during the month of January, 1866, are given as illustrative of the application of thermometry in disease; together with an abstract of the highly interesting and elaborate paper of Dr. L. Thomas, of Leipzig, on the thermal phenomena of pneumonia. ("Ueber die Temperatur Verhaltnisse bei crouposer Pneumonie," Archiv der Heilkunde; Bd. V, S. 30–36.)

The cases are accompanied by a diagram, *fac simile* of the tables of "Vital Signs," used at the bedside to make the daily record of temperature, pulse-beats and respirations. This one only differs from ours in that on it are represented the curves for three cases, whereas usually but one case is put upon a table. No further explication of the diagram is necessary, except to state that the heat is registered in decimal parts of degrees; the pulse and respirations, of course, by whole numbers.

Two observations are found in each daily column, one in the morning made at 9.10 A. M., one for the latter part of the day made at 4.15 P. M.

For want of space the cases are much abbreviated; the physical examinations being nearly omitted. This may be justified by stating that the diagnosis in each case was verified by the attending physician, Dr. Wm. H. Draper, and that careful daily examinations were made by Dr. J. Haven Emerson, the talented house physician of the Hospital.

CASE I. G., æt. 28. Steamship fireman.

Admitted Jan. 11th, 1866. Was taken ill on 6th with chill, pain in right side, etc.

On admission, considerable febrile disturbance of rather low type; tongue much coated, inclined to dry in middle; general condition fair. Physical examination reveals dulness, bronchial breathing and voice, and crepitant rale over limits of right upper lobe; most marked in supra-spinous fossa. Expectoration characteristic. Is ordered decubitus, an oiled silk jacket, at night a Dover's powder, and

℞. Pulv. ipecac, gr. ij.
Liq. ammon. acetat., ℥iv.
M. Cap. ℥ss. q. 4. h.

Jan. 12. Consolidation progressing; expectoration thin, containing some pure blood; had epistaxis yesterday; tongue rather dryish and brown coated. Ordered, Sherry wine, ℥ vi, to be taken with milk; cont. mixt.

Jan. 15. Defervescence occurred during last twenty-four hours, with a fall of six degrees. (See table.) General bronchitis has supervened, and masks the physical signs in the affected lobe. Tongue is quite moist and cleaner. Stop mixt.

Jan. 17. Quite convalescent. Ordered, continue wine and take quinine sulph., gr. ij, t. i. d.

Jan. 27. For a week has sat up and been about. Bronchitis is gone, and he is discharged cured.

CASE II. R. S., æt. 26. Seaman.

Admitted Jan. 16th, 1866, having been ill four days. Sickness began with chill, pain in right side, fever and cough.

On admission he presents all the objective signs of pneumonia, involving the right lower lobe, in stage of red hepatization; expectoration rust-colored; general condition good. Ordered, decubitus, at night min. xv of Squibb's liq. opii co., and

℞. Pulv. ipecac, gr. ij.
Liq. ammon. acetat., ℥iv.
M. Cap. ℥ss. q. 4. h.

An oiled silk jacket, lined with cotton, was applied over chest.

Jan. 21. Fall in evening temperature indicates the limitation of the disease. A pleuritic friction sound is heard at angle of scapula. Cont. treatment.

Jan. 23. Yesterday complete defervescence did not occur, because the inflammation extended to the upper lobe of same side; tympanitic dullness is found over angle of scapula. Defervescence did occur this A. M. Yesterday, patient had a little epistaxis and diarrhœa, which latter was checked by suppos. Opii, (gr. ij.) p. r. n. Expectoration less colored, more abundant and muco-purulent. Cont. treatment.

Jan. 24. Vital signs down to normal standard.

Jan. 31. Resolution took place rapidly; patient has been up for a few days. Some dullness remains at apex and over lower lobe, and at angle of scapula are still heard pleuritic friction sound, some subcrepitant rale and broncho-vesicular breathing.

Feb. 5. Patient is discharged, cured.

Case III. S., æt. 19. Coal-passer on steamship.

Admitted Jan. 25th, 1866. Illness began on 21st with chill, pain at lower ribs of right side, cough and fever.

On admission there is much pain, great febrile movement and dyspnœa; expectoration rust-colored, very characteristic; patient presents all physical signs of inflammation involving the right upper lobe. General condition excellent, except previous malarial poisoning. Ordered, an oiled silk jacket, a hypodermic injection of morphia (min. x of Magendie's sol.) and mixture used in cases above, in same doses. Decubitus.

Jan. 27. There is complete hepatization of affected lobe. Defervescence occurred during last twelve hours, with a fall of six and a half degrees. Had epistaxis yesterday. Cont. treatment.

Jan. 29. Subcrepitant rale (rale crepitant redux) is heard over upper lobe; expectoration muco-purulent, hardly at all colored; no fever. Stop mixt.

February. Patient convalesced very rapidly, and only remains in hospital for treatment of malarial element.

RECORD OF VITAL SIGNS.

DAYS OF DISEASE 5 6 7 8 9 10 6 7 8 9 10 11 12 4 5 6 7

DEGREES OF FAHRENHEIT.

108 107 106 105 104 103 102 101 100 99 98

PULSE-BEATS PER MINUTE.

140 130 120 110 100 90 80 70 60

RESPIRATIONS

50 45 40 35 30 25 20

MERRILL SC.

In a few words these three cases may be summed up.

But first, the word "defervescence" used in these histories and in the abstract below needs definition. It is new to American medical literature, having first been used by the learned professors of the German schools, to express in one word the cessation or subsidence of the febrile phenomena of disease. It has been very recently adopted in England, and finds a prominent place, as well as the entire subject of thermometry, in Aitken's "Science and Art of Medicine."

In reading the cases and looking over the table of vital signs, the first thing to be remarked is, at what a late period of the disease the patients were received; the most recent on the fourth day. This, however, is a difficulty which attends all investigations made upon hospital subjects; but, fortunately, in other places the disease has been studied so early as to determine very conclusively that the increase of fever is rapid, and that a very high temperature may be expected within the first twelve hours. In cases I and III, which were typical of simple acute inflammation of one lobe, the temperature, high at the the beginning of the observation, continued to rise, or did not fall except in so far as the regular morning remissions were concerned, until a certain point of the disease had been reached, the ninth day in one case and the fifth in the other, and not till the ascending pathological changes had been wrought; in other words, not until hepatization of the implicated lobe was fully established. This fall in heat, pulse and respiratory movements (defervescence) was complete in case III, the thermometer never again rising above 98.° In case I, the same was true, with the exception of a single moderate elevation in the evening of the tenth day. Case II was remarkable for the low intensity of the fever. The defervescence was partially effected on the ninth and tenth days, but during the evening of the latter an alarming increase ot 3° led us to suspect that the disease had invaded a second lobe, when it will be noticed that the pulse and respirations (80 and 30 respectively) gave no warning. Physical examination showed that the thermometer was right. The next morning defervescence occurred fully, and finally in the course of two days. It may also be seen how accurately the estimation of the heat determines the cessation of ascending pathological changes, and marks the beginning of those processes by which the vital actions restore the parts affected, and the system generally, to a state of health. That the third period of pneumonia is a resolution, is confirmed by the fact that, in normal cases, the temperature never rises above 99° after hepatization is complete. Were it true that this disease ends by a suppurative stage, the thermometer would doubtless remain high until the process was completed.

In these two points the diagnosis of disease and of complications, and reliability as an element of prognosis, lies the great value of this means of observation. To go into all the facts necessary to sustain these positions, would require more space than can be allowed to a hospital report intended simply to call attention to, and invite trial of, the matter in question. Suffice it to say, that Prof. Wunderlich, of Leipzig, has, with others, so thoroughly investigated the subject before giving it to the profession, that he made no less than half a million careful observations, and ascertained the temperature-variations of nearly all diseases so accurately, that his pupils can, by merely looking at the diagramatic record of a case, almost always correctly diagnosticate the disease without having seen the patient or heard of any other symptoms. He and many other leading physicians make constant use of the thermometer in private practice. The limited trial made in this Hospital has not at all lowered the expectations raised by reading the published accounts; in many cases a diagnosis has been arrived at, and a complication detected long before the other objective signs would have enabled us so to do. More especially are the distinctions of fevers, into the great types of typhus, typhoid, remittent and intermittent, clearly indicated and not to be mistaken. A last point urged is, that the surface heat, as measured in the axilla, is not liable to variations from the nervous, emotional causes which render the pulse and respiration so very changeable and unreliable. Being the direct result of the molecular changes produced by pyrexia, (although the precise relation of the degree of heat to the amount and proportions of the substances, resulting from retrograde metamorphosis, is not yet ascertained,) it cannot be immediately affected by causes acting through the senses, which so disturb other objective signs, for instance, the sudden arrival of the physician, of a friend, of news, the movements frequently necessary to the comfort of the patient, or to facilitate examination, etc.

The whole matter of the utility of medical thermometry is founded upon the fact, that the normal temperature of the human body is invariably fixed within certain limits. Very numerous observations by competent observers have determined this. The following are those of Prof. Traube, of Berlin; the average of many studies of healthy adults at fixed periods of the day, taken in axilla:

| | | |
|---|---|---|
| 98.24° | at 7 A. M. | |
| 98.69° | at 10 A. M. | After breakfast. |
| 98.65° | at 1 P. M. | |
| 98.78° | at 5 P. M. | After dinner. |
| 98.24° | at 7 P. M. | |

As to daily practice with the thermometer, the instrument should be an accurately made one, perfect in every respect. Those used abroad (not at present to be had in this country) are graduated to fifths and tenths of degrees, and should be preferred. However, one graduated to degrees only will suffice, if greater care be exercised in reading off the mercury, and a practiced eye may even estimate one-fourth of a degree on such a scale. In all cases the thermometer should have an outer glass casing, to protect it from injury and external influences.

The bulb is to be inserted in the axilla, just beneath the fold of the pectoralis major muscle, not too deeply, the forearm of that side carried across the chest, and the elbow secured by an assistant, or by the patient's other hand. It is left *in situ*, carefully isolated from all clothing, and in perfect contact with the skin, for eight or even ten minutes, being looked at three or four times, the last two determining whether the column of mercury has ceased to rise; the degree (and fraction) is then read off and registered. While waiting, the physician has time to count and record the pulse and respirations, and even to proceed with many other points of investigation. If time be precious, the bulb may previously be heated about to the expected heat, and then inserted, when three or five minutes will be enough for a correct estimate. With the exception of the anus, the axilla is found to be the most reliable locality for the purpose.

For further details and general information, the reader is referred to the *Archiv der Heilkunde*, of Leipzig; to Aitken's *Science and Art of Medicine*, and to articles in the London *Medical Times and Gazette*, for 1858 and 1861.

(ABSTRACT.)

*Early observations:*

| | | | | |
|---|---|---|---|---|
| 102.5° | observed | 4 | hours after | first symptoms. |
| 104.9° | " | 9 | " | " |
| 104.4° | " | 12 | " | " |
| 105.1° | " | 23 | " | " |
| 105.8° | " | 36 | " | " |
| 106.5° | " | 24 | " | " |

*Variations of temperature in regular cases:*

Minimum in morning, maximum in afternoon, and again after midnight a decrease.

*Types of fever:*

1st. In no case purely continued.

2d. Some cases with small differences between the morning and evening measurements, (°.45 to °.5,) have very seldom been observed, and only for a single day.

3rd. In the vast majority of cases the differences amounted to °.9 to 2°.

4th. More than once differences of 2.5° have been noted.

5th. A few cases in which almost complete remissions occurred.

The *highest point of fever* may be known by an extraordinary elevation, or by a great fall in the temperature, compared with preceding observations.

The *termination of the fever* (defervescence) generally occurs within forty-eight hours, the temperature sinking to the normal standard. The *days of defervescence* have been carefully noted.

46 CASES BY THOMAS.

| Days. | Cases. |
|---|---|
| 2d, | 2 |
| 3d, | 6 |
| 4th, | 6 |
| 5th, | 11 |
| 6th, | 5 |
| 7th, | 10 |
| 8th, | 4 |
| 9th, | 0 |
| 10th, | 2 |

107 CASES BY ZIEMSSEN.

| Days. | Cases. |
|---|---|
| 2d, | 0 |
| 3rd, | 9 |
| 4th, | 3 |
| 5th, | 31 |
| 6th, | 5 |
| 7th, | 35 |
| 8th, | 4 |
| 9th, | 9 |
| 11th, | 8 |
| 13th, | 3 |

Thus out of a total of 153 cases, 45 turned on the seventh day and 42 on the fifth. Ziemssen states that in cases in which more than one lobe is involved, the defervescence is likely to be put off until the eleventh or thirteenth day. Thomas is, however, inclined to doubt this, for in 18 of his cases, in which more than one lobe was affected, he observed but two in which the pyrexia did not subside by the seventh day; and those on the tenth.

Defervescence is affected by the lobes involved as follows:

Right upper lobe—1 on second day; 4 on seventh; 1 on eighth.
Right lower lobe—2 on fourth; 4 on fifth; 2 on sixth; 1 each on seventh, eighth and tenth.
Left lower lobe—1 on second; 5 on third; 2 on fourth; 6 on fifth; 1 on eighth.

*Temperature towards fatal termination.*

Majority of cases ending in delirium:

2 on sixth day, 106.5° and 105.6.°
1 on seventh, 109.4.°
2 on eighth, 106.4° and 104.9.°
1 on fourteenth, 108.9.°

In one case ending with furious delirium, hallucinations, trismus and tetanus, an elevation of 6.9° occurred in six hours. In three cases dying of suffocation, (asthenia and apnœa,) there was no elevation toward the agony; in two there was great emphysema, and the temperature remained between 101.3° and 103.5°; in the third, accompanied by a fever of great regular remissions, the last estimate was 99.5.°

## Figures and Tables

**Figure f1:**